# Center of rotation restoration and acetabular cup sizing in total hip arthroplasty: the predictive role of radiographic femoral head diameter. A single-center study from the FP-UCBM hip study group

**DOI:** 10.3389/fsurg.2026.1931094

**Published:** 2026-09-08

**Authors:** Biagio Zampogna, Alessandro Del Monaco, Francesco Rosario Parisi, Michele Paciotti, Andrea Zampoli, Augusto Ferrini, Giovanni Zagaria, Antongiulio Manfreda, Ferruccio Vorini, Rocco Papalia

**Affiliations:** 1 Operative Research Unit of Orthopaedic and Trauma Surgery, Fondazione Policlinico Universitario Campus Bio-Medico, Rome, Italy; 2 Research Unit of Orthopaedic and Trauma Surgery, Department of Medicine and Surgery, Università Campus Bio-Medico di Roma, Rome, Italy; 3 BIOMORF Department of Biomedical, Dental, Morphological and Functional Images, University of Messina, Department of Orthopaedic and Trauma Surgery, A.O.U. Policlinico “G. Martino”, Messina, Italy

**Keywords:** acetabular cup size, center of rotation, component position, COR, femoral head diameter, radiographic templating, THA, total hip arthroplasty

## Abstract

**Purpose:**

Restoring the center of rotation (COR) in total hip arthroplasty (THA) is essential. We hypothesized that relatively smaller cups are associated with COR displacement. Our aim is to evaluate how acetabular cup size (ACS), relative to the radiographically measured femoral head diameter (FHD), correlates with COR positioning in THA.

**Methods:**

We retrospectively analyzed 251 patients undergoing unilateral THA using a posterolateral approach with a non-arthritic contralateral hip that was used to assess FHD on preoperative x-ray. From postoperative radiographs, cranial cup displacement (cCD) and medial cup displacement (mCD) relative to contralateral hip COR were measured. Patients were divided into three groups based on the difference between ACS and FHD (*Δ*): Group A (*Δ*≤2.5 mm), Group B (2.5 mm<*Δ*<8.5 mm), and Group C (*Δ*≥8.5 mm). Patients with a cCD <3 mm and mCD <5 mm were included in the “Restored COR” group; the others in the “Non-Restored COR” group.

**Results:**

Mean cCD and mCD values were: 3.93 ± 4.29 mm and 5.13 ± 4.13 mm (Group A), 2.14 ± 3.25 mm and 2.56 ± 3.28 mm (Group B), 2.41 ± 2.75 mm and 2.06 ± 3.09 mm (Group C); Group A showed significantly higher cCD and mCD than Groups B and C (*p* < 0.001). In Group A, the relative risk (RR) of non-restored COR was 1.55 (95% CI 1.26–1.91; *p* < 0.001) compared to group B + C. Patients with Restored COR (46.6%) had lower FHD than patients with Non-Restored COR (53.4%).

**Conclusion:**

COR restoration in THA is influenced by cup size. The implant of cups with ACS ≤ FHD+2.5 mm is associated with a significantly increased risk of COR displacement, especially in patients with larger FHD.

**Level of evidence:**

III (retrospective comparative cohort study).

## Introduction

Total hip arthroplasty (THA) is a successful procedure for hip osteoarthritis (1), aiming to relieve pain, restore joint function, and improve quality of life (2).

Accurate restoration of the center of rotation (COR) in THA is essential for proper biomechanical reconstruction, influencing muscle function, stability, and range of motion (ROM) (3–6).

One of the most commonly used approaches in the literature for estimating correct radiographic positioning and sizing of the acetabular cup has been the Ranawat triangle method (7–9). However, several studies have shown significant deviations from the anatomical center of rotation using this method (10, 11).

More recently, the concept of restoring the native hip COR has been introduced as a reference parameter for correct cup positioning (4, 12–14). COR restoration, and thus conservation of native acetabular position, offers several advantages: preservation of acetabular bone stock, reduction of the risk of bony or soft tissue impingement, improved range of motion and reduced risk of dislocation, improved abduction, enhanced patient outcomes, reduced wear, and lower long-term risk of loosening (15, 16). To improve COR restoration, peripheral acetabular reaming has been proposed as a more effective technique than the “conventional” one (12, 15), resulting in the use of larger cup diameters, which appear closely correlated with COR position.

Several studies have shown that radiographic or intraoperative femoral head measurement can be a good predictor of acetabular cup size, though without assessing the accuracy of COR restoration (17–20). Moreover, both measurement methods may be difficult to perform in cases of deformity, osteophytes, or residual cartilage, whereas the contralateral healthy hip can be reliably used for preoperative planning in THA (21).

This study aims to evaluate the correlation between radiographically measured femoral head size and both the size and COR position of the implanted acetabular cup.

## Materials and methods

The current retrospective study was approved by the local ethics committee that received approval on 31 May 2023, with protocol number PAR 93.23 OSS. according to the principles of the Declaration of Helsinki. The study included only consecutive patients who underwent unilateral THA between April 2020 and June 2023 at our institution. Inclusion criteria: unilateral end-stage hip osteoarthritis, pre- and postoperative digital anteroposterior (AP) radiographs of the pelvis, and complete clinical data. Exclusion criteria: cemented or revision THA, secondary osteoarthritis (dysplasia (22), post-traumatic, epiphysiolysis, dysmetabolic disorders), and severe contralateral osteoarthritis (Kellgren–Lawrence III–IV). Patients whose radiographs showed pelvic misalignment (23) were also excluded. Preoperative sociodemographic and clinical data were collected. Acetabular cup size (ACS) for each implant was extracted from the operative records.

### Surgical procedure

Cementless porous stems with uncemented hemispherical press-fit acetabular cups were used for all patients. In just six cases, additional fixation with two acetabular screws was performed after definitive press-fit seating of the cup had been achieved. In all cases, the bearing surface consisted of ceramic on highly cross-linked polyethylene. All cases were performed in the same institution by three expert surgeons using a minimally invasive posterolateral approach in lateral decubitus. A traditional (“conventional”) progressive reaming technique (12) was used for acetabular preparation: it started with a small-diameter reamer, typically 39 mm, and progressively increased in size until adequate circumferential contact and satisfactory press-fit stability with the acetabular bone were achieved. A preoperative digital planning was performed using standard AP radiographs for each replacement to estimate component sizing and the expected restoration of biomechanical parameters.

### Imaging analysis

Standardized pre- and postoperative AP pelvic radiographs were obtained following established protocols (23). Radiological parameters were evaluated using Materialise OrthoView® (Materialise, Belgium). On preoperative radiographs, the native femoral head diameter (FHD) was measured on the contralateral healthy side. A best-fit circle was adapted to the radiographic contour of the native femoral head, and its diameter was recorded as the FHD. The contour was defined on the osseous profile of the femoral head. Postoperatively, the prosthetic (pCOR) and native (nCOR) centers of rotation were defined as the centers of circles fitted to the acetabular cup and contralateral femoral head. Horizontal and vertical COR positions were measured as previously described (15). Medial cup displacement (mCD) was the difference between nCOR and pCOR horizontal positions; cranial cup displacement (cCD) was the difference between vertical positions. Positive values indicated medial or cranial shifts of the pCOR. The cut-off values of <3 mm for cCD and <5 mm for mCD were chosen to define COR restoration, based on previous studies that demonstrated that exceeding these limits may negatively affect hip biomechanics and stability (24). Leg length discrepancy (LLD) was assessed pre- and postoperatively. All radiographic measurements were independently performed by two orthopedic senior residents trained in musculoskeletal imaging analysis. Intra- and inter-observer reliability was evaluated in a random group of 50 patients, resulting in intraclass correlation coefficients (ICC) of 0.93 and 0.88, respectively.

### Radiological outcomes

**Figure 1:**
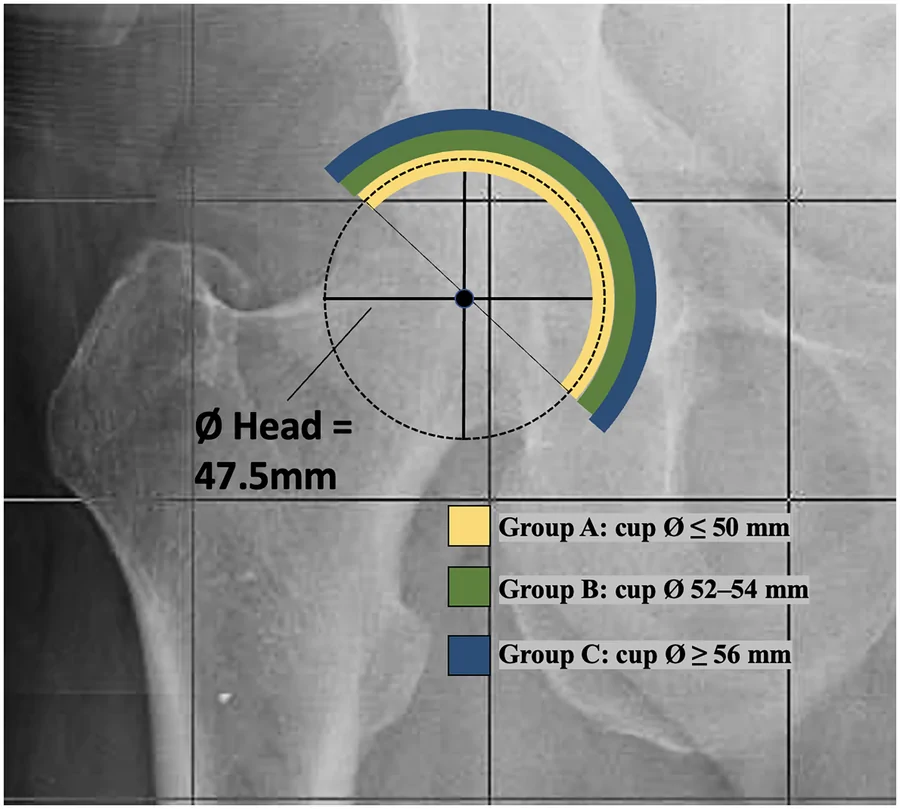
Graphical example of the difference between acetabular cup size and femoral head diameter (*Δ*) used for group stratification.

For each patient, the difference between the ACS and the FHD (*Δ*) was calculated to assess the mismatch between the prosthetic component and native anatomy. Patients were divided into three groups according to the value of *Δ*: Group A included patients with *Δ* ≤ 2.5 mm; Group B included those with 2.5 mm < *Δ* < 8.5 mm and Group C included patients with *Δ* ≥ 8.5 mm (Figure 1).

The difference in COR positioning between these groups was evaluated. A 2.5 mm threshold was selected to approximate the native femoral head diameter, correcting for cartilage thickness (∼1.5 mm per side, ∼3 mm total) (25–27) and a 0.5 mm error related to the limited accuracy of radiographic measurements. An 8.5 mm threshold was set to define “large” cups, derived from a + 6 mm oversizing limit associated with increased postoperative pain risk (28), plus 2.5 mm for cartilage thickness and radiographic correction. An additional stratification was made between patients with a Restored COR (R), defined as those with both mCD < 5 mm and cCD < 3 mm, and those with a Non-Restored COR (NR).

### Statistical analysis

Statistical analysis was performed using R software. Patients were stratified by *Δ* value (Groups A, B, C) and COR restoration (Restored, R; Non-Restored, NR). Multivariate normality of the biomechanical outcome variables was assessed using the Henze–Zirkler test. Global differences among Groups A, B, and C were evaluated using MANOVA with Pillai's Trace. For multiple comparisons, Bonferroni correction was applied, with an adjusted significance threshold of *α* = 0.0167. Welch's *t*-test was used to compare FHD between the R and NR groups, whereas differences in FHD among Groups A, B, and C were assessed using one-way ANOVA followed by Tukey's *post-hoc* test. Associations between *Δ* groups and COR restoration status were assessed using the chi-square (*χ*^2^) test or Fisher's exact test, as appropriate. Relative risks (RRs), with 95% confidence intervals (CIs), were calculated to estimate the risk of COR non-restoration. Statistical significance was set at *p* < 0.05.

## Results

A total of 251 consecutive patients undergoing primary unilateral cementless THA with a posterolateral approach were included according to inclusion and exclusion criteria. Cohort characteristics are shown in Table 1.

**Table 1:** Demographic and clinical characteristics of the study population (*n* = 251).

Variable	*n*	Mean ± SD	Range
Age	251	65.3 ± 12.4	23–89
Female	143 (57%)		
Male	108 (43%)		
BMI (kg/m^2^)	251	27.5 ± 4.5	18.4–43.2
Femoral head diameter (mm)	251	45.7 ± 3.8	37.0–56.0
Acetabular cup size (mm)	251	52.5 ± 3.1	48.0–62.0
*Δ* (mm)	251	6.8 ± 5.0	−7.0–21.5

Stratification based on *Δ* showed the following patients distribution: 51 (20.3%) in Group A (*Δ* ≤ 2.5 mm), 113 (45.0%) in Group B (2.5 mm < *Δ* < 8.5 mm), and 87 (34.7%) in Group C (*Δ* ≥ 8.5 mm).

Mean cCD was significantly higher in Group A compared to Group B and C (Table 2).

**Table 2:** Comparative analysis of biomechanical parameters and COR restoration by *Δ* group.

Parameter		Group A (*n* = 51)	Group B (*n* = 113)	Group C (*n* = 87)	*p*-value
Cranial Cup Displacement (cCD)	Mean ± SD	3.93 ± 4.29	2.14 ± 3.25	2.41 ± 2.75	<0.001
	95% CI	2.72–5.13	1.53–2.74	1.83–3.00	
Medial Cup Displacement (mCD)	Mean ± SD	**5.13** ± 4.13	2.56 ± 3.28	2.06 ± 3.09	<0.001
	95% CI	3.96–6.29	1.95–3.17	1.40–2.71	
Femoral Head Diameter (FHD)		50.46 ± 2.53	45.80 ± 2.94	42.80 ± 2.35	<0.001
Restored COR	117 (total)	13	56	48	
Non-Restored COR	134 (total)	38	57	39	
Absolute Risk of COR Non-Restoration		74.5%	50.4%	44.8%	*p* = 0.002

**Figure 2:**
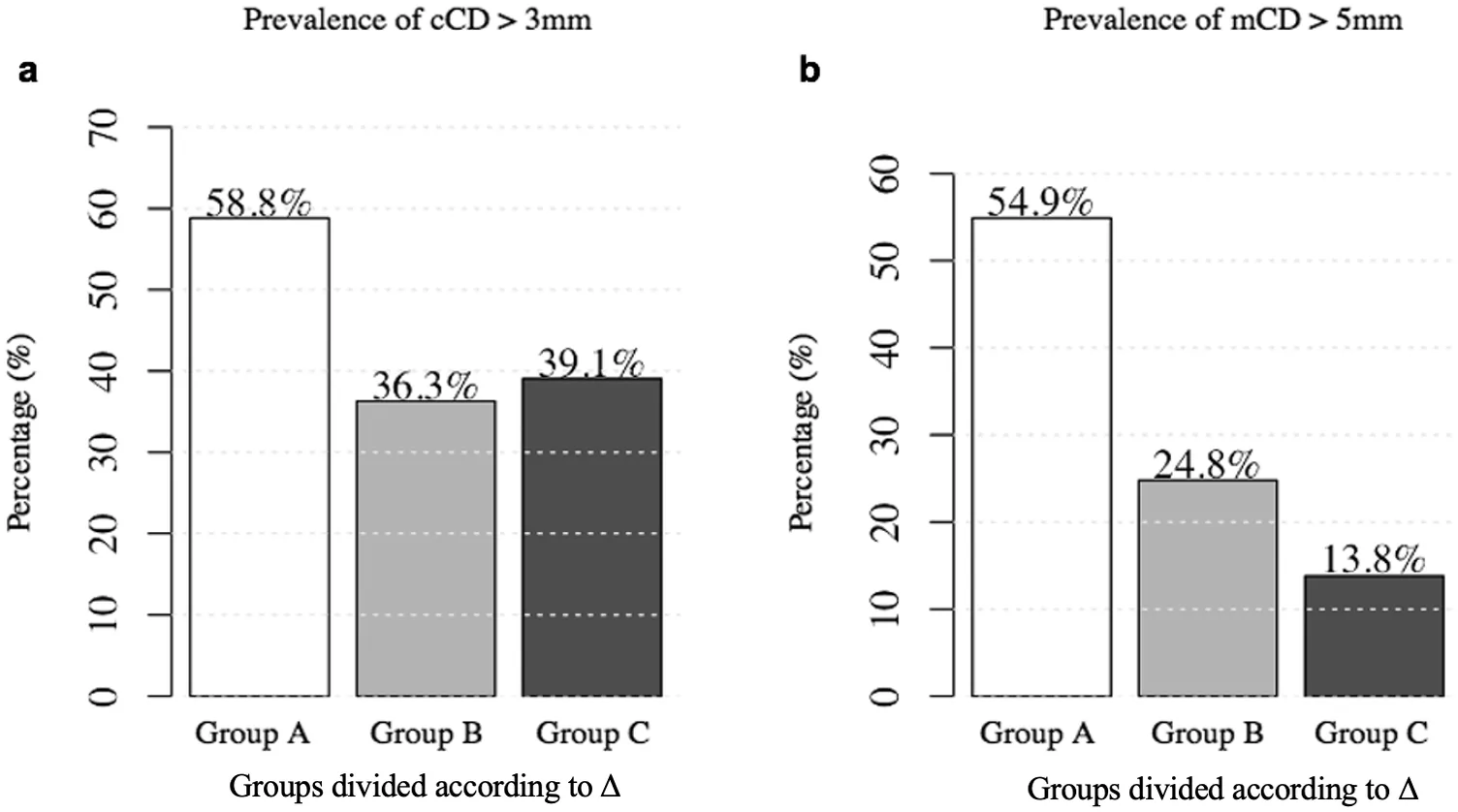
(**a**) Prevalence of cCD>3 mm by *Δ* group: Group A 58.8%, Group B 36.3%, Group C 39.1%. (**b**) Prevalence of mCD>5 mm by *Δ* Group: Group A 54.9%, Group B 24.8%, Group C 13.8%.

In Group A, the proportion of patients with cCD exceeding the 3 mm threshold was 58.8%, clearly higher than in Groups B and C, at 36.3% and 39.1%, respectively (Figure 2a). Mean mCD was significantly higher in Group A compared to Groups B and C (Table 2). In Group A, the percentage of patients with mCD exceeding the 5 mm threshold was 54.9%, compared to 24.8% in Group B and 13.8% in Group C (Figure 2b).

**Figure 3:**
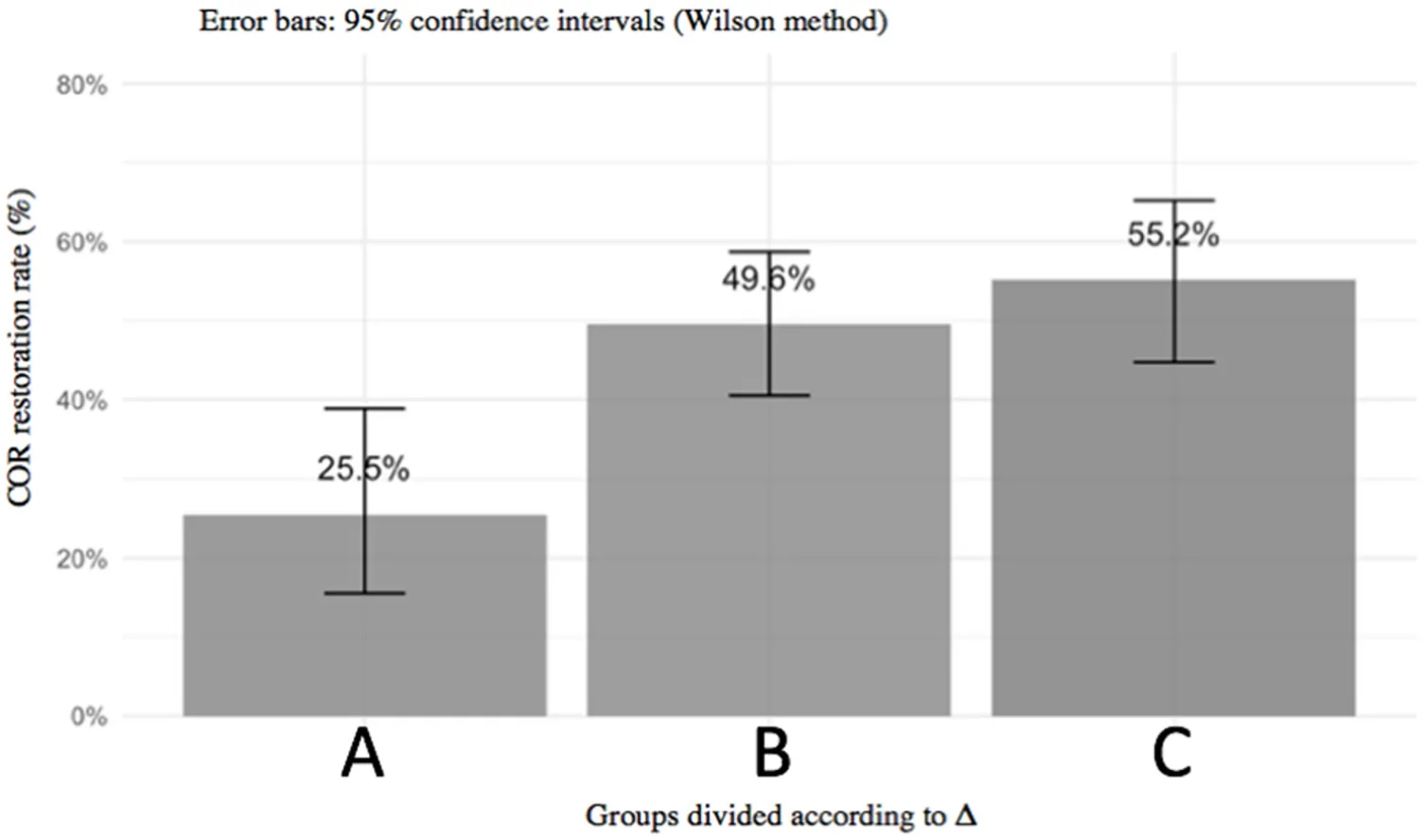
COR restoration rate across *Δ* groups: group A 25.5%, Group B 49.6%, Group C 55.2% (*p* = 0.002).

Based on the stratification by COR restoration (cCD < 3 mm and mCD < 5 mm), 117 patients (46.6%) were included in the R (Restored COR) group, 134 patients (53.4%) in the NR (Non-Restored COR) group. The analysis of absolute and relative risk of failed COR restoration revealed significant differences among the *Δ*-based subgroups (Tables 2, 3, Figure 3): in Group A, nearly 3 out of 4 patients (74.5%) did not achieve adequate restoration, compared with about 1 in 2 patients in Groups B (50.4%) and C (44.8%). The lack of risk difference between Groups B and C shows that exceeding the 8.5 mm threshold does not further reduce malpositioning (Table 3; Figure 3).

**Table 3:** Relative Risk of Center of Rotation (COR) Non-Restoration Across ***Δ*** groups. Relative Risk Increase (RRI) is calculated as (RR−1) × 100.

Comparison	Relative risk (RR)	Relative risk increase (RRI) %	*p*-value
Group A vs. B	1.48 (95% CI: 1.18–1.85)	48%	<0.001
Group A vs. C	1.66 (95% CI: 1.29–2.14)	66%	<0.001
Group B vs. C	1.13 (95% CI: 0.86–1.47)	13%	0.43
Group A vs. B + C	1.55 (95% CI: 1.26–1.91)	55%	<0.001

Comparative analysis showed a significant FHD difference between Restored and Non-Restored COR groups: 44.73 ± 3.34 mm (median 44.0) vs. 46.57 ± 4.01 mm (median 46.5) *p* < 0.001. ANOVA showed highly significant FHD differences among Groups A, B, and C (*p* < 0.001) (Table 2) showing an inverse pattern: larger FHDs corresponded to smaller *Δ* values and vice versa.

## Discussion

Our findings highlight acetabular cup size (ACS) and native radiographic femoral head diameter (FHD) as key variables influencing the accuracy of COR restoration. We identified a 2.5 mm threshold for the difference between ACS and FHD (*Δ*): below this value (Group A), COR restoration was inadequate in about 75% of patients; above this cutoff, the risk of malposition decreased to ∼50%, supporting the use of cups larger than FHD + 2.5 mm as a practical target.

We observed an oversizing plateau effect: beyond 8.5 mm, no further COR restoration benefits were seen, suggesting that excessive bone removal for very large cups may be unnecessary (28).

The native radiographic FHD emerged as a factor associated with COR non-restoration: patients in the Non-Restored COR group had significantly larger native FHD than those in the Restored COR group. A larger FHD may reflect a more voluminous acetabular anatomy, potentially providing greater room for unintended medialization or cranialization during reaming.

Conversely, in patients with smaller FHD, acetabular bone stock is limited with less margin for deviation from the native COR. Furthermore, cups smaller than size 46 are rarely implanted, as they do not allow for a satisfactory head-neck ratio. The importance of restoring the COR has been extensively studied in recent years, and it has been shown to influence muscle function, stability, ROM, and implant longevity. Although the Ranawat triangle method has been widely used historically to define the correct positioning of the acetabular cup (7–9), several studies have questioned its reliability reporting a tendency to locate the COR more cranially and medially than the anatomical center measured on the contralateral healthy hip (10, 11).

Kurtz et al. (4) showed that COR medialization and cranialization reduce Range of Motion Before Impingement (ROMBI), not fully compensated by femoral adjustments, altering biomechanics and increasing dislocation risk. Cranial positioning of the COR weakens the function of the abductor muscles (3). García-Rey et al. (5) found that non-anatomical COR reconstruction with poor abductor restoration raises dislocation risk even respecting the “safe zones”. Timperley et al. (13) further argued that there is no true “safe zone” for version and inclination unless native biomechanics and COR are preserved.

To more accurately replicate the patient's native COR, various surgical techniques for acetabular preparation have been proposed including navigation, robotics, or alternative manual reaming (12, 15, 29, 30). Bonnin et al. (12) and Meermans et al. (15) demonstrated that peripheral (or “anatomical”) reaming achieved minimal COR displacement (mCD 0.8 mm, cCD 0.7 mm) and larger cup diameter compared with conventional reaming (mCD 5 mm, cCD 3.7 mm). The choice of one technique over another not only influences the ability to restore COR but also has a direct impact on the diameter of the implanted acetabular cup.

In this study, we investigated how cup size relates to COR positioning and whether it can serve as a marker of its alteration. Previous studies have focused on predicting cup size from FHD without considering COR restoration (17–20). Odri et al. (28) reported that cups exceeding the intraoperatively measured FHD by more than 6 mm increases the risk of persistent postoperative pain.

To our knowledge, no study has investigated the relationship between FHD and ACS in COR restoration, nor has a lower threshold for cup selection been identified. Undersized cups not only raise the likelihood of failing to restore the COR, but also require smaller femoral heads, thereby limiting ROM and increasing the risk of dislocation due to a shorter jumping distance (31). Our data show that implanting smaller acetabular cups, defined as ACS ≤ FHD + 2.5 mm (Group A), was associated with a 1.55-fold higher risk of COR non-restoration compared with Groups B and C combined (RR 1.55, 95% CI 1.26–1.91; *p* < 0.001). As a consequence, the use of a relatively small cup that achieves an apparently satisfactory fit during reaming can be considered a practical intraoperative sign of potential cranial or medial displacement of the COR. Being aware of this risk allows for adjustment of the reaming technique or serves as a valid warning signal to perform an intraoperative radiograph to detect any shift and differentiate cranial from medial displacement, thereby guiding compensation at the femoral level by increasing height or offset. Furthermore, this cutoff helps to preoperatively define the cup size, which should be approximately 3–8 mm larger than femoral head diameter. Our analysis revealed larger FHDs among non-restored cases, suggesting that error is more frequent with larger heads.

This study has limitations. Its retrospective, single-center design limits control of confounders and generalizability. All procedures were performed using a posterolateral approach, a conventional reaming technique, and a uniform strategy based on combined anteversion and a femur-first technique, which enhances consistency but restricts applicability to other implant philosophies. COR migration may also be influenced by reaming direction, depth, and unrecognized bone defects, factors not assessed here. Radiographic measurements of the contralateral femoral head may not fully represent intraoperative anatomy due to side-to-side differences. In our opinion, the proposed threshold may also be applicable for cup sizing in patients with bilateral disease when femoral head sphericity is preserved; however, it has not been specifically validated in this population and requires further investigation, potentially using CT-based measurements.

In conclusion, this study highlights the importance of considering the size of the implanted acetabular cup for accurate restoration of the COR in THA. Implanting cups with a diameter less than or equal to the radiographic FHD plus 2.5 mm significantly increases the risk of medial and cranial displacement of the COR. Furthermore, the *Δ* value (ACS–FHD) provides a preoperative and intraoperative indicator that may help guide acetabular preparation and enhance surgical accuracy.

## Data Availability

The raw data supporting the conclusions of this article will be made available by the authors, without undue reservation.
